# P-761. Antibiotic Prescribing Patterns among Outpatients with Acute Cystitis in Michigan

**DOI:** 10.1093/ofid/ofaf695.972

**Published:** 2026-01-11

**Authors:** Allison Frazer, Elisia Stier, Anne Haddad, Shruthi Degala, Brenda M Brennan, Adam J Scott, Anurag Malani, jason M Pogue

**Affiliations:** University of Michigan College of Pharmacy, Ann Arbor, MI; Michigan Department of Health and Human Services, Lansing, Michigan; Michigan Department of Health and Human Services, Lansing, Michigan; Michigan Department of Health and Human Services, Lansing, Michigan; Michigan Department of Health and Human Services, Lansing, Michigan; University of Texas - Austin, Ann Arbor, Michigan; Trinity Health Michigan, Ann Arbor, Michigan; University of Michigan, College of Pharmacy, Ann Arbor, MI

## Abstract

**Background:**

Acute uncomplicated cystitis is a common illness in the outpatient setting requiring antibiotic therapy. While there are multiple treatment options for cystitis, some are preferred for antimicrobial stewardship (AMS) purposes, and outpatient prescribing practices are mostly unknown. This study aimed to describe antibiotic prescribing for uncomplicated cystitis among outpatients in Michigan.

**Figure 1:**
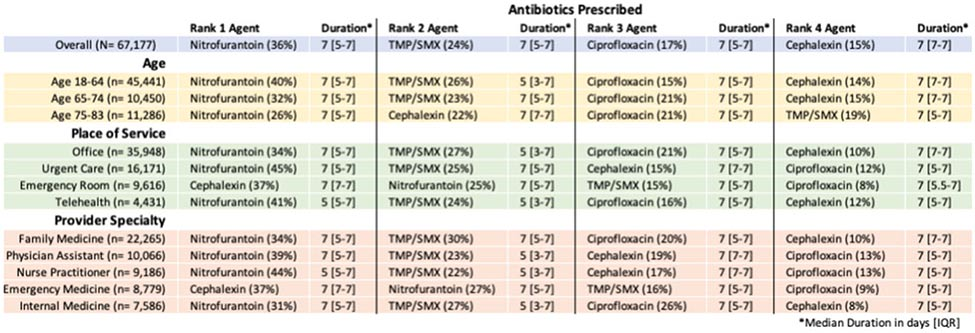
Agent Selection

**Figure 2:**
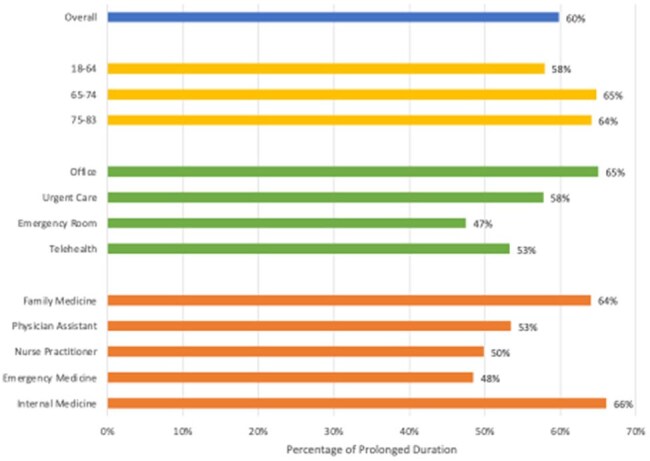
Frequency of prescriptions with durations longer than guideline recommendation by different subgroups in Michigan

**Methods:**

The Michigan Department of Health and Human Services received medical and pharmacy claims data from IQVIA from 2019 – 2021. Outpatient females ≥ 18 years of age with medical claims data with a diagnosis code for acute cystitis (N30.00 and N30.01) and a corresponding antibiotic prescription were included. Prescribing patterns of antibiotics (agent selection, dose, and duration of therapy) were analyzed for the overall cohort and stratified by age, place of service, provider specialty, and patient zip code.

**Figure 3:**
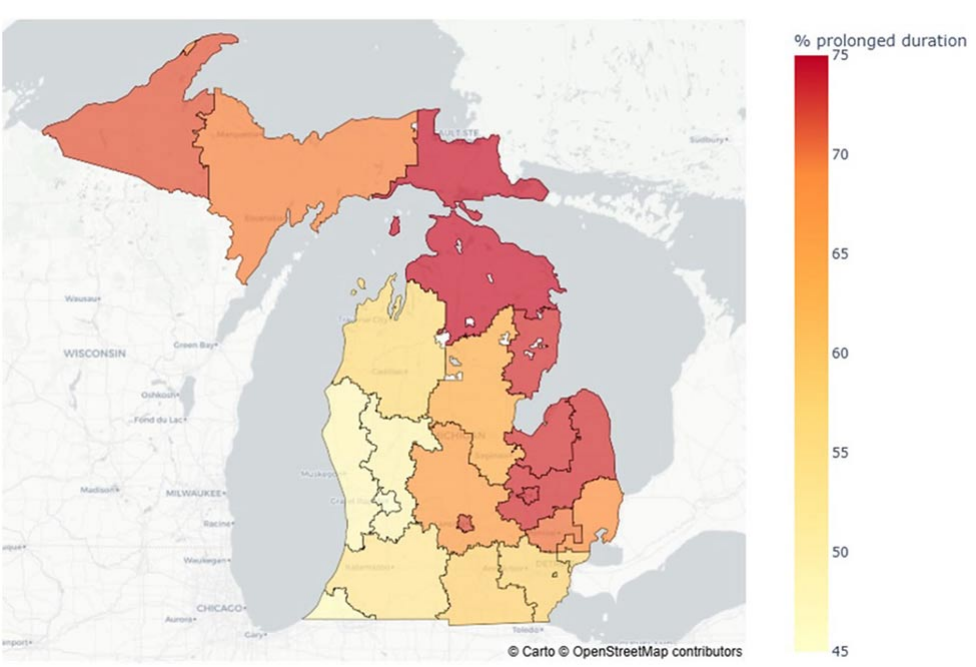
Frequency of prescriptions with durations longer than guideline recommendation by patient zip code in Michigan

**Results:**

From 2019 - 2021 there were 67,177 unique encounters from 57,513 patients for acute cystitis. The most prescribed antibiotics were nitrofurantoin (36%), trimethoprim-sulfamethoxazole (TMP-SMX, 24%), ciprofloxacin (17%), and cephalexin (15%), making up a total of 93% of all antibiotic prescriptions. 60% of prescriptions for these agents were for durations longer than guideline recommendations. Prolonged durations were most common with ciprofloxacin prescriptions (85%) and least with cephalexin (17%). Prescribing practices were largely consistent across place of service, provider specialty, patient zip code, and age group, with prolonged durations of therapy prescribed more frequently in patients ≥ 65 years of age, treated at an office visit, and treated by a family or internal medicine provider (Figures 1, 2). Additionally, geographical variation in frequency of prolonged durations of therapy was observed (Figure 3). A notable shift to cephalexin prescribing was also seen in patients treated in the emergency room.

**Conclusion:**

The study revealed high prescribing rates of TMP-SMX and ciprofloxacin in Michigan, as well as prolonged durations of therapy. These findings were consistent in subgroups analyzed and emphasize the need to prioritize outpatient AMS practices for acute cystitis.

**Disclosures:**

jason M. Pogue, PharmD, Entasis: Advisor/Consultant|Entasis: Grant/Research Support|GlaxoSmithKline: Advisor/Consultant|Melinta: Grant/Research Support|Merck: Advisor/Consultant|Merck: Grant/Research Support|Shionogi: Advisor/Consultant|Shionogi: Grant/Research Support

